# Maximal mouth opening in infants and toddlers with spinal muscular atrophy: a prospective controlled study

**DOI:** 10.1186/s13023-024-03524-z

**Published:** 2025-01-15

**Authors:** Jana Zang, Deike Weiss, Charlotte Dumitrascu, Julia Glinzer, Marie Wegner, Anna Strube, Jonas Denecke, Almut Niessen, Christina Pflug

**Affiliations:** 1 Department of Voice, Speech and Hearing Disorders, University Dysphagia Center, University Medical Center Hamburg-Eppendorf, Hamburg, Germany; 2 Institute of Health Sciences, University of Luebeck, Lübeck, Germany; 3 Department of Pediatrics, University Medical Center Hamburg-Eppendorf, Hamburg, Germany; 4 Insitute of Product Development and Mechanical Engineering Design, Hamburg University of Technology, Hamburg, Germany

**Keywords:** Spinal muscular atrophy, Jaw opening, Bulbar function

## Abstract

**Background:**

Bulbar function is frequently impaired in patients with spinal muscular atrophy (SMA). Although extremely important for the patient’s quality of life, it is difficult to address therapeutically. Due to bulbar dysfunction, maximum mouth opening (MMO) is suspected to be reduced in children with SMA. However, no published MMO values exist for SMA children younger than 24 months. This study presents a novel approach to measuring MMO in infants and toddlers with SMA and compares it with healthy controls.

**Methods:**

Children with SMA (0–24 months) who received disease-modifying therapy at a single neuropediatric center and similarly aged healthy children were prospectively recruited. MMO was measured using a cardboard scale and a custom-designed instrument.

**Results:**

A total of 115 children were included (SMA = 24, healthy controls = 91). Inter-rater reliability between two examiners was excellent (ICC = 0.987, 95% CI 0.959 to 0.995), as was the reliability between the cardboard scale and the custom-designed instrument (ICC = 0.986, 95% CI 0.968 to 0.994). A mixed linear model showed a significant increase of MMO with age, and a significantly wider mouth opening in healthy controls (*p* < .001).

**Conclusion:**

For future research, MMO can provide valuable information about the involvement of cranial nerves, particularly in the context of disease-modifying therapies, even at a very early age.

**Supplementary Information:**

The online version contains supplementary material available at 10.1186/s13023-024-03524-z.

## Introduction

Spinal muscular atrophy (SMA) is a rare neurodegenerative disease due to a homozygous deletion or mutation of the survival motor neuron (*SMN*1) gene, resulting in decreased *SMN* protein production [1, 2]. The most common form is the clinically classified SMA type 1, usually associated with one to three copies of the *SMN*2 gene [3]. Children with untreated SMA type 1 exhibit severe muscle weakness and fail to reach any motor milestones. Abnormal bulbar function results in problems with swallowing, often requiring tube feeding. Three medications have been approved for treating SMA, leading to longer survival, improved motor function, and achievement of motor milestones. However, bulbar function remains impaired in some patients despite treatment [4–6]. In SMA, maximal mouth opening (MMO) has long been considered a significant indicator of the impairment of the bulbar cranial nerve nuclei [7–9]. A good MMO is associated with a better outcome for dysphagia, choking, and mastication [7, 9, 10]. On the other hand, a very advanced reduction in jaw opening is a serious problem for intubation (e.g., during operations) [10].

Until recently, research and therapy studies have mainly focused on motor function, particularly the motor neurons, front horn cells, and spinal canal. However, there is a growing interest in the swallowing and the bulbar symptoms of SMA in early childhood [11–13].

Studies with untreated and late-treated cohorts have shown that MMO differs significantly between SMA types [14] or between sitters and walkers [7]. As the disease progresses, MMO is thought to decrease [7]. In MRI studies, Wadman and colleagues [10] demonstrated that this decrease is associated with fatty degeneration of the lateral pterygoid muscle, which negatively impacts the anterior sliding movement (condylar sliding) during mouth opening. Mouth opening requires the coordinated action of several muscles and nerves. The key muscle groups involved besides the temporomandibular joint are the digastric muscle, the mylohyoid muscle, the geniohyoid muscle, and the lateral pterygoid muscle. To achieve the maximum possible mouth opening, the rotation of the condyle is followed by an anterior sliding, which allows for further lowering of the mandible. Muscles responsible for mouth closing, like the masseter muscle, were found to be less affected in the study by Wadmann et al. [10]. Based on their findings on mandibular function in adult patients, van Bruggen and colleagues [15] confirm that mouth opening involving the lateral pterygoid muscle is notably affected in these patients. In contrast, bite force—which relies on the masseter and temporalis muscles—remains stable. The authors suggest that certain muscles may be more vulnerable than others.

In adults with SMA type II or III, the compound muscle action potential (CMAP) of the facial nerve, along with the number and size of motor units in the orbicularis oris muscle, was found to be significantly lower compared to healthy controls. Both CMAP and the number and size of motor units were found to correlate with MMO [16].

Another approach is the measurement of intraoral pressure, e.g. with the Iowa Oral Performance Instrument (IOPI) [7, 17]. Colot and colleagues [17] used IOPI with children starting at the age of three and demonstrated a significant difference in terms of maximum lip-, tongue-, and masseter pressure in a sample of 20 treated children with SMA (aged 3.5–16.5 years) when compared to a healthy control group (*n* = 53).

A ruler was mainly used to assess MMO in patients with different types of SMA [7, 8, 10, 14, 16]. One study used the TheraBite^®^ ROM Scale [9] as an alternative. Data on the development of MMO in infancy, particularly in children with two *SMN*2 copies or clinical symptoms of SMA type I, are not yet available.

Two studies have assessed MMO in healthy children under two years of age. Nowak and Casamassimo [18] measured mouth opening in 422 children aged six weeks to 36 months. They used the TheraBite^®^ Range of Motion (ROM) Scale by opening the mouths of the children in a “scissor-like” motion. If no teeth were present, the alveolar crest distance (ACD) was measured; if teeth were present, the incisal edge distance (IED) was measured.

A more recent study by Ahmadi and colleagues [19] examined 151 healthy infants and toddlers aged zero to eleven months (mean age 5.2 months). The “distance between the occlusal edges of the alveolar ridges” was measured with a plastic ruler (mm) by placing the thumb and forefinger in the child’s mouth and applying slight pressure to open it. For infants with erupted central incisors, the gum edges of the incisors were used as the landmark. The authors discussed that children under three years of age are more prone to measurement errors due to reduced cooperation.

A wide range of MMO within age groups for healthy children is reported [19, 20]. While no gender differences were found for young children [19, 21], Müller and colleagues [20] were able to demonstrate significant differences between boys and girls from the age of 13 years onwards for a cohort of 20 719 children (10 060 girls, 10 659 boys).

This study aimed (1) to provide measurement of MMO in infants and toddlers in the first two years of life, (2) to record the first MMO values in a sample with SMA at that age, and (3) to compare MMO with healthy controls.

Additionally, a quick and accurate measuring instrument was developed for MMO measurement in infants and small children to overcome issues with the previously used methods.

## Methods

The measurement of MMO is a subproject of the OSMA study (objective capture of swallow-related outcomes in infants and young children with SMA) registered with the German Clinical Trials Register (DRKS00032287, WHO registry, https://trialsearch.who.int/Trial2.aspx?TrialID=DRKS00032287, 14 Juli 2023). The local ethics committee has approved the study (2022-100827_2-BO-ff). All parents have given written informed consent.

### Sample

Children younger than 24 months diagnosed with genetically confirmed SMA and two or three *SMN*2 copies were eligible for the study. All received any of the three currently available therapies at a single neuropediatric center between September 2021 and September 2024. No stricter inclusion criteria were defined for the study, meaning that children detected through newborn screening and those with clinical diagnosis were included. This sample is part of the ongoing DySMA study (Dysphagia in Children with Spinal Muscular Atrophy) [22]. The routine recording of MMO in this cohort started in September 2022, and the focus study OSMA was initiated in July 2023. Multiple measurements were taken over a maximum of 18 months after the start of therapy.

As part of the ongoing DySMA-norm study, a healthy control group was recruited between April 2023 and March 2024 in Northern Germany. The recruitment process involved maternity clinics, playgroups, and daycare units. To be included in the study, children needed to be born after 37 weeks of gestation with no developmental abnormalities. Only one examination was conducted per child, but the sample was selected for a homogeneous age distribution between zero and 24 months to represent developmental trajectories based on ascending age groups.

### Material

**Fig. 1:**
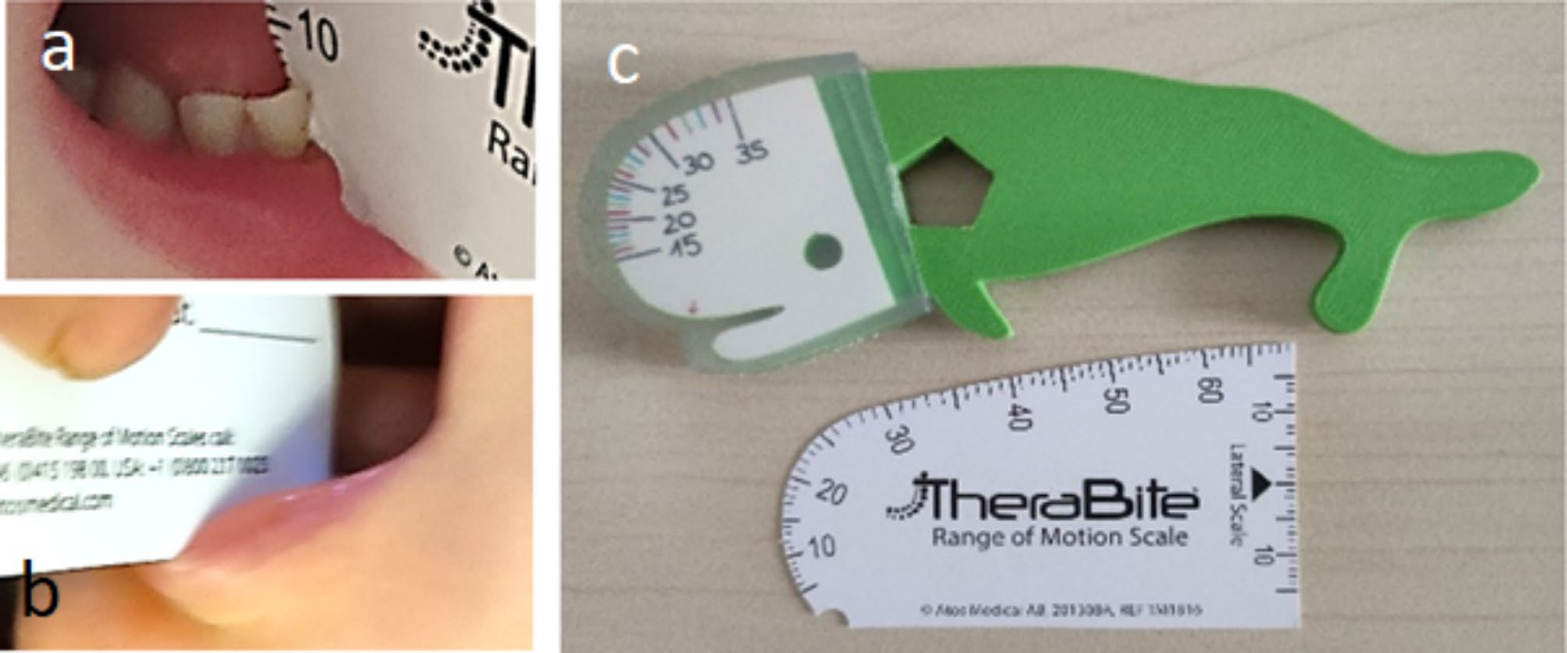
Issues with using cardboard scale in toothless or gap-toothed infants: **a**) slipping into the gaps between the teeth, softening of the material, **b**) cutting into the lip, and being able to read only from one side, **c**) designed and manufactured whale and TheraBite^®^ ROM Scale

Routine measurement of MMO was carried out using a cardboard scale (TheraBite^®^ ROM, see Fig. 1c). However, during pilot testing, some issues occurred: the cardboard scale tended to press into the gums or slip between the teeth, and in toothless or gap-toothed infants, it became soft. Furthermore, the card could only be read from one side (Fig. 1a, b). Measurement with a ruler was difficult or impossible without great effort and required significant intervention in the infant’s mouth. In newborns, the sucking reflex and tongue protrusion were particularly problematic during all trials.

To address these problems, a measuring scale was developed in collaboration with the Institute of Product Development and Mechanical Engineering Design (PKT) that should be particularly suitable for toothless infants, easy to use, and appealing to small children. The development process followed the classical process outlined in the VDI 2221 [23]. The measuring scale should suit the small oral cavity and cover a measuring range of up to 35 mm. The scale should provide quick and precise readings and be easy to use, with a secure grip for the clinician. After creating several prototypes, the shape of a whale was chosen for the measuring scale. The core of the whale was printed using an UltiMaker 5 S 3D printer (UltiMaker B.V., Geldermalsen, the Netherlands) with a thermoplastic polyester filament (PLActive) from the manufacturer Copper 3D (Copper 3D, Chile). The material is classified as food-safe by the FDA and EU and is recommended for medical applications, prostheses, or surgical aids.

The whale-shaped measuring scale has a reference edge marked by a red arrow that should be placed on the lower alveolar ridge and then moved into the mouth to define the starting point (see Fig. 1c). The measuring range on both sides covers jaw openings from 15 to 35 mm in one-millimeter increments. It has different bold colors, making it easy to read. The measuring area is covered with a cast silicone cover to prevent incision (see Fig. 1c) and has small notches to read off.

### Implementation

**Fig. 2:**
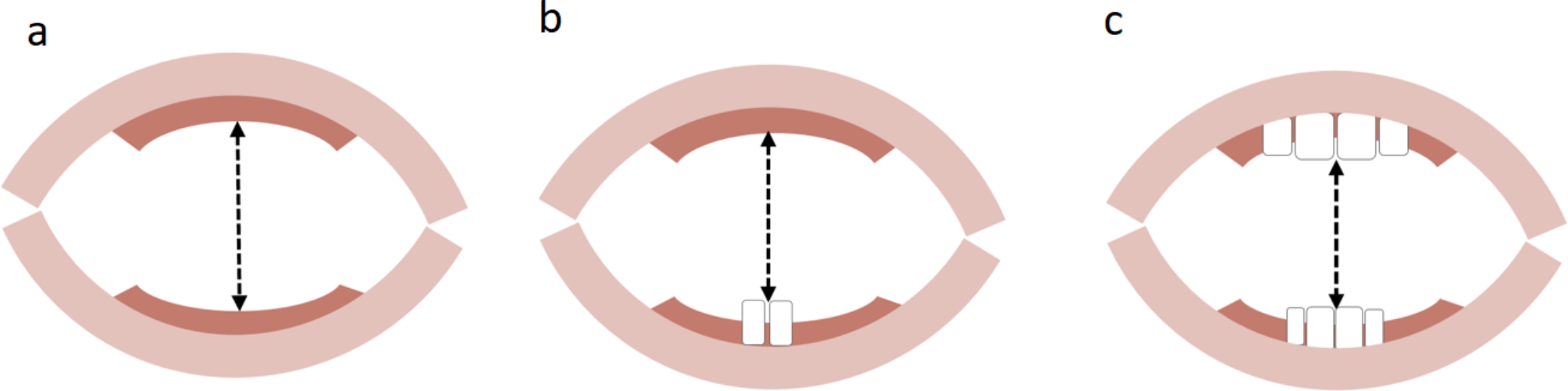
Measuring MMO: the distance between **a**) alveolar ridges, **b**) incisors and alveolar ridges, and **c**) between incisors

The measurement was taken using either the cardboard scale, the whale, or both in a manner appropriate for the age and situation. The actual opening of the mouth was measured and defined as MMO. In infants without teeth this was the distance between the alveolar ridges (see Fig. 2a). In children with teeth, the distance between the incisors was measured (see Fig. 2c). In cases where only the upper or lower jaw had teeth, the distance between the alveolar ridge on one side and the incisor on the other side was measured (see Fig. 2b). The term “teeth” was used when the teeth had already erupted beyond the jaw ridges, protruding out of the gums.

The measurement was carried out identically in children with SMA and healthy controls and was always performed by one out of two possible speech-language pathologists (SLP). Children with SMA were examined during routine neuropediatric check-ups in a clinical setting; healthy controls were tested at home, in the playgroup, or the clinic, depending on recruitment. Newborns and infants usually lay on their backs or in their parent’s arms. Toddlers sat on their parent’s lap, in strollers, on a chair, or on the floor. An experimental procedure was used to initiate mouth opening, where parents were encouraged to contribute their ideas. The measuring scales were always ready for spontaneous use during a playing session or other assessments.

The following methods were frequently used to stimulate mouth opening: (a) Triggering rooting in hungry infants, (b) gently pressing down the chin, (c) waiting for yawning or laughing, (d) offering a pacifier or food next to the scale, and (e) demonstrating the measurement with the parents or a puppet and motivating the child to imitate it.

The measurement was carried out within one to two seconds without any pressure to force the jaw open. One measurement per instrument was carried out and evaluated. After the measurement, the parents were asked to rate whether they found the measured MMO realistic. The measurement was repeated if the parents stated the child could open their mouth much wider. If both measuring instruments were used for reliability (cardboard scale and whale), the value of the cardboard scale was always used as the officially recorded score.

To determine inter-rater reliability, two SLPs participated together in 12% of the sessions. One took the measurement, and both read the value and noted the sore.

### Data Analysis

All data were tabulated in Microsoft Excel (2016) and imported into SPSS (Version 27, IBM) for statistical analysis. To calculate inter-rater reliability between both conducting SLPs, the intra-class correlation (ICC) was computed using a two-way mixed-effects model with absolute agreement for two raters, along with 95% confidence intervals (CI). The interpretation of ICC was based on Koo and Lee [24]: >0.5 poor; between 0.5 and 0.75 moderate; between 0.75 and 0.9 good; > 0.90 excellent reliability.

The reliability between the TheraBite^®^ ROM Scale and the whale was also calculated by ICC (two-way mixed-effects model, absolute agreement, multiple measurements). A Bland-Altman plot was created to visualize the agreement between the two measuring scales.

Data obtained from the samples were analyzed and presented descriptively, reporting mean and standard deviation (SD). In addition, median and interquartile range (IQR) were reported due to non-normal distributed data in both samples. MMO for both groups was visualized using scatterplots.

For further statistical analysis, all children were categorized into age groups, defined as follows: 1 = 0–2 months, 2 = 3–5 months, 3 = 6–8 months, 4 = 9–11 months, 5 = 12–14 months, 6 = 15–17 months, and 7 = 18–24 months.

To compare the differences between all children with SMA and healthy controls (HC) a mixed linear model was conducted with MMO as the dependent variable, case ID as the random intercept, while age group served as the random slope. The group (0 = HC; 1 = SMA) and age group (1–7) were included as fixed effects.

For comparisons within the SMA group, the S*MN2* copy number (2 or 3) and age group were used as fixed effects. The level of significance was set to 0.05.

## Results

### MMO in children with SMA and healthy controls

The parents of 25 children with SMA and 92 healthy controls agreed to have their children participate in the study. In all cases, measurements were carried out without applying any pressure. However, one child with SMA repeatedly refused measurement at the ages of eleven, 17, and 24 months. Similarly, one child from the healthy control group, aged 14 months, also refused to participate in the examination. This resulted in data from 24 children with SMA and 91 healthy controls. In the SMA sample, 15 children had two *SMN2* copies, and nine had three *SMN2* copies. None of the children with three *SMN2* copies had dysphagia; all were fed orally, and no tongue fasciculations occurred during the study period in these children. Among the children with two *SMN2* copies, two were fed via nasogastric tube or, from the age of 12 or 14 months, respectively, via percutaneous endoscopic gastrostomy due to severe dysphagia. These two children were already symptomatic before the start of medical therapy. The group with two *SMN2* copies showed tongue fasciculations at an average age of ten months, with two children younger than six months during the study period and one child who did not have any tongue fasciculations even at 19 months. Sample characteristics are summarized in Table 1.

**Table 1:** Sample characteristics

	Healthy controls (*n* = 91)	SMA total (*n* = 24)	SMA 2 *SMN2* (*n* = 15)	SMA 3 SMN2 (*n* = 9)
Sex girls: boys	34:57	13:11	9:6	4:5
Age range at MMO measurement in months	0–23	0–26*	0–26	0–19
Identified through NBS (n)	-	22	15	7
Median age at start medication in days (IQR)	-	27.5 (17)	28.0 (16)	27.0 (216)
Presymptomatic start of medication (n)	-	18	11	7
Type of medication (n)				
onasemnogene nusinersen risdiplam risdiplam followed by onasemnogene	-	19 2 1 2	12 1 1 1	7 1 0 1
Type of nutrition (n)				
exclusively oral tube-feeding	91 0	22 2	13 2	9 0

Note IQR = interquartile range, NBS = newborn screening. *Due to scheduling difficulties, three of the children with SMA were already 25, and 26 months old at the time of the last examination

**Fig. 3:**
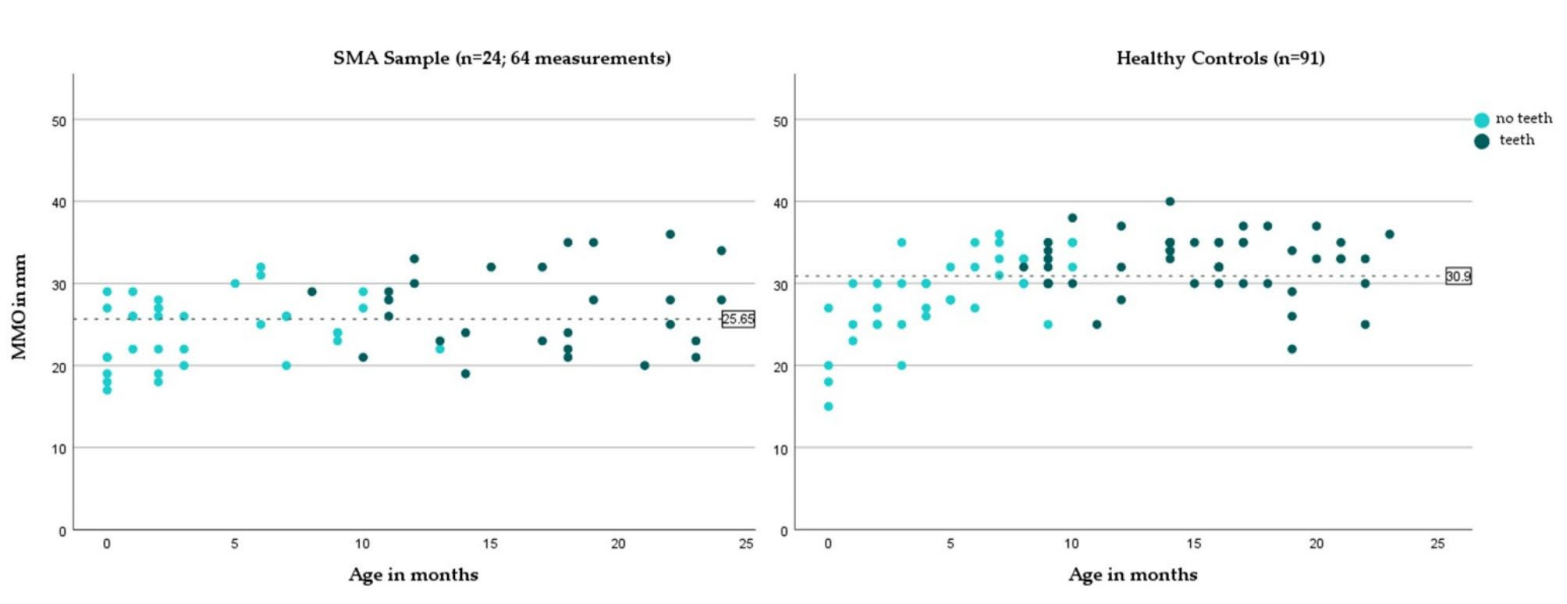
Scatterplot of MMO values for the SMA sample and healthy controls. Toothless children and children with teeth are shown in different colors

In the group of children with SMA, all 24 children had repeated examinations, resulting in 64 measurements. The mean age for all measurements was 9.9 months ± 7.84. The children were toothless in 36 cases. The first teeth in the SMA group started to appear at the age of ten months. The mean MMO was 25.66 mm ± 4.62. MMO in children with teeth (27.11 mm ± 4.05) was higher than without teeth (24.5 mm ± 3.93) which represents an increase with age (Fig. 3). A difference between children with two and three *SMN2* copies was particularly evident in the group of children with teeth (see Table 2).

**Table 2:** MMO values

	Healthy controls		SMA total			SMA (2 SMN2)		SMA (3 SMN2)	
	n*		n*			n*		n*	
	M (SD)	Mdn (IQR)	M (SD)	Mdn (IQR)		M (SD)	Mdn (IQR)	M (SD)	Mdn (IQR)
	41		36			23		13	
no teeth	28.9 (4.97)	30.0 (8)	24.5 (3.96)	26.0 (6)		24.39 (4.17)	26.0 (7)	24.8 (3.70)	26.0 (6)
	50		28			17		11	
teeth	32.7 (3.55)	33.0 (5)	27.11 (5.05)	28.0 (9)		25.12 (4.87)	23.0 (8)	30.18 (3.73)	29.0 (5)
	91		64			40		24	
total	30.9 (4.60)	32.0 (7)	25.66 (4.62)	26.0 (7)		24.7 (4.43)	24.5 (7)	27.3 (4.56)	27.5 (9)

Note Mdn = median, IQR = interquartile range, *M* = Mean, SD = standard deviation. Median and mean are both reported due to non-normal distribution in both groups. *n here corresponds to the number of measurements

For the healthy controls, the group of 91 children corresponded to 91 examinations. Out of these, 41 children were toothless. The first teeth began to appear when the children were eight months old. The mean age of the healthy controls was also 10.5 months ± 6.36. The mean MMO was 30.9 mm ± 4.60. Among the toothless children, the mean MMO was lower at 28.9 ± 4.97 than the mean MMO of children with teeth (M = 32.7 ± 3.55). Medians and IQR for the MMO in both samples are reported in Table 2. A detailed list of MMO values ​​for all age groups can be found in supplementary Table S1.

The mixed linear model included a total of 155 MMO examinations in 115 children (HC *n* = 91; SMA *n* = 24). The MMO showed a significant increase with age (*p* < .001), and the healthy controls exhibited a significantly wider mouth opening than children with SMA.

Within the SMA group, there was also an increase with age (*p* = .024), though this increase was somewhat lower. However, no significant difference in mouth opening was observed between the groups with two and three SMN copies (Table 3).

**Table 3:** Mixed linear models

	Between HC and SMA			Within SMA		
Fixed effects	Estimate^a^	95% CI^b^	p-value	Estimate^a^	95% CI^b^	p-value
Group (0HC/1SMA)	-5.06	-6.56 - -3.56	< 0.001	-	-	-
Age group (1–7)	1.03	0.65 − 1.40	< 0.001	0.70	0.10 − 1.30	0.024
SMNCoNr (2/3)	-	-	-	1.76	− 0.47 − 4.00	0.114

Note Dependent variable = MMO, random intercept = Case ID, random slope = Age group, ^a^Estimate=regression coefficient, ^b^95% CI = confidence interval of the regression coefficient; (a) HC = healthy controls includes a total of 91 participants resulting in 91 measures; SMA = all children with SMA, includes a total of 24 participants resulting in 64 measures. (b) SMNCoNr = number of SMN2 copies, which can be two or three. The ordinal variable “Agegroup” is treated as a continuous factor under the assumption of linearity, resulting in a single estimate that reflects the average change in MMO per unit increase in “Agegroup”

### Reliability

**Fig. 4:**
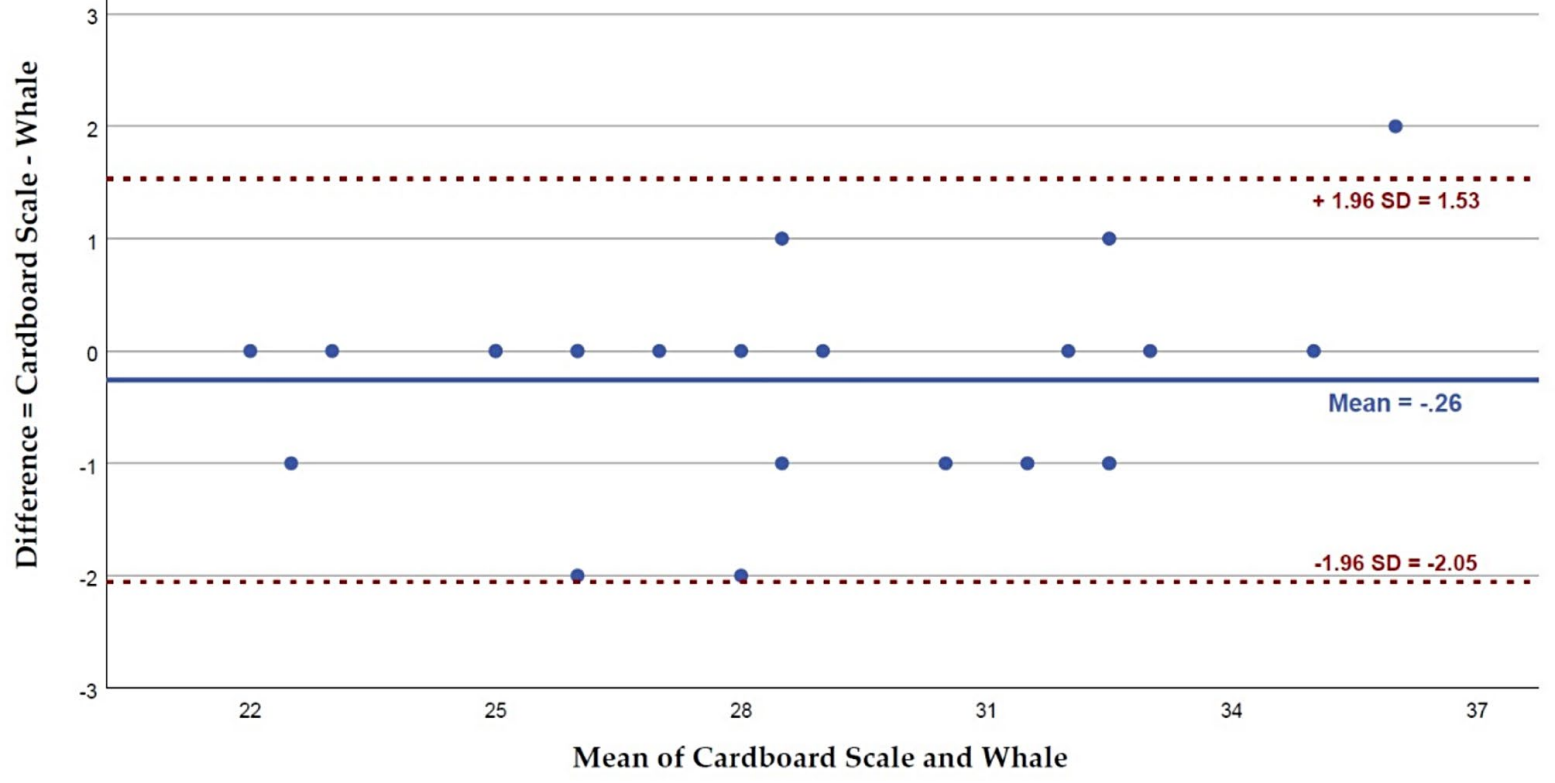
Bland-Altman plot for differences in measuring MMO with TheraBite^®^ ROM Scale and Whale in 23 cases. Mean difference (bias) = − 0.26 mm; agreement ranges from − 2.05 to 1.53 mm

Out of the 155 measurements, 18 were rated by both examiners in the same session. Their inter-rater reliability was excellent, with ICC = 0.987 (95% CI 0.959 to 0.995). Similarly, based on 23 sessions where both scales were used on the same child, the reliability between the cardboard scale and the whale was also excellent with ICC = 0.986 (95% CI 0.968 to 0.994). The Bland-Altman plot shows a mean difference of − 0.26 mm (95% CI − 0.260 to 0.135) between both instruments, with an agreement range between − 2.05 and 1.53 mm (Fig. 4).

## Discussion

Our study shows that the measurement of MMO can be reliably and accurately carried out in children with and without SMA during their first two years of life. Our findings indicate that mean MMO in children with SMA is significantly lower than in a healthy control group and that children with three *SMN2* copies tend to be less affected by reduced MMO at this age compared to those with two *SMN2* copies. In addition, the newly developed whale measuring scale showed excellent agreement with the conventional cardboard scale (mean difference of − 0.26 mm) and is therefore suitable for measuring up to 35 mm MMO. The scale has been designed to overcome the challenges of the cardboard scale, preventing cutting into the child’s mouth allowing for easy, quick, and accurate reading from both sides while being visually appealing to small children. As children with SMA often undergo unpleasant examinations, our gentle, child-friendly method allows for repeated examinations to track developmental progress. The new device was accepted by all children but two (one with SMA, one healthy child).

Assessing the MMO in infants and toddlers can be challenging for a few reasons. Firstly, they are initially toothless, which makes it impossible to measure the distance between their incisors. Secondly, they cannot yet actively follow the request to open their mouth as wide as possible. Due to their limited compliance, obtaining normative data on healthy young children through multiple measurements is not always possible. In general, it can be confirmed that measuring MMO in children of this age is challenging, and doing so requires creativity and patience.

MMO values in healthy children have only been published by Ahmadi and colleagues at ages zero to eleven months [19] and by Nowak and Casamassimo for ages six weeks to 24 months [18]. Our sample of toothless healthy controls (*n* = 41) showed a mean MMO of 28.8 mm (95% CI 27.31 to 30.45), which is lower than Ahmadi and colleagues’ sample (*n* = 151) with a mean value of 32.1 mm (95% CI 31.5 to 32.7). The difference in measurement methods (ruler vs. cardboard scale or whale) or the gentler approach, which avoids passive pressure, could explain these differences. Calculating the mean MMO value for the toothless children from Nowak and Casamassimo’s study, where the same cardboard scale was used (*n* = 123), results in 29.4 mm, which corresponds better to our results. Comparing the mean MMO value of 30.9 mm (95% CI 29.94 to 31.86) for all healthy controls regardless of dentition status (*n* = 91) with the calculated mean value for all children aged zero to 24 months from the study by Nowak and Casamassimo (*n* = 300, M = 32.8 mm) shows slightly lower results for our sample. The distribution of our data could play a role here, as our medians are closer to the means of Nowak and Casamassimo’s study. The lower values could also be explained by the, on average, slightly older sample of Nowak and Casamassimo (six weeks to 24 months). In both our samples and the standard values reported in other studies, we observe an initial increase during the first few months of life, followed by a slower progression from the eighths month onward. This pattern has been linked to changes associated with teething and growth. During this period, the jaw continues to grow while the teeth are still erupting. In the group of children with SMA, particularly those with two *SMN2* copies, stagnation of MMO is visible. This stagnation may be due to disease progression, which at this age is typically accompanied by the first appearance of tongue fasciculations, even when early treatment has been initiated. In summary, it can be assumed that measurements in this study were carried out well and align with the values of larger samples.

Previous studies demonstrated that MMO differs between SMA types in older children and adults with SMA [7, 14]. In those studies, patients with SMA type 2 had a reduced MMO compared to patients with SMA type 3. Accordingly, patients with three *SMN2* copies were more severely affected than patients with four *SMN2* copies. Both studies were conducted in children five years and older as well as adults. Our data suggest that reduced MMO occurs much earlier. In our sample, MMO was already impaired in early infancy, especially in children with two *SMN2* copies.

It is important to highlight that there is an overlap in the distributions for all groups, which is particularly evident when examining the values by age group (see supplementary Table S1). This overlap arises from the fact that there is generally a wide range of MMO among all children, including those with significant outliers in the SMA groups. Overall, these values must always be interpreted in the context of the children’s developmental stages. Unlike adult patients, young children with SMA can experience both an increase in MMO due to development and growth and a decrease as the disease progresses. Therefore, longitudinal studies examining children beyond the age of 24 months are particularly valuable. A more comprehensive discussion of this topic in relation to adult patients, which extends beyond our study, can be found in the work of van Bruggen and colleagues (2024) [15].

It can be assumed that MMO is an important marker indicating that bulbar symptoms and cranial nerve involvement occur at a very early stage of SMA. This is further evidenced by the early occurrence of tongue fasciculations in children with two *SMN2* copies or clinically diagnosed SMA type 1 [11, 25, 26]. How the reduced MMO can ultimately be treated or what prevention looks like is still unclear. Increased attention is needed here in the future. Observational studies should pursue approaches to prevent the reduced MMO in early infancy and focus more on the entire bulbar symptomatology in SMA.

### Limitations and considerations for future studies

As this is a single-center study and SMA is a rare disease, a sample size of 24 affected children is considered relatively large. However, the sample size is still insufficient for comparing symptomatic and presymptomatic treatment initiation outcomes. The association between low MMO and dysphagia could not be statistically analyzed because only two children with dysphagia were included.

It is expected that with very early and ideally presymptomatic therapy, there will be fewer cases of early-onset and severe dysphagia in the future. A longer observation period and multicenter pooling of samples or analysis of patient registries could be considered to obtain more accurate results.

Our raw data indicate that children with three *SMN2* copies had a larger MMO and no tongue fasciculations during the study period. Again, the sample size is too small to draw conclusions using inferential statistics. The reliability analysis, in particular, should be critically viewed. Koo and Lee [24] recommend at least 30 ratings and three raters to calculate the ICC. There were 18 measurements for the inter-rater reliability and 23 for the comparison between the instruments. The small number of reliability measurements resulted from organizational and personnel reasons. It can also be criticized that one examiner used the instrument for inter-rater reliability, and both examiners read off the value. However, pilot tests showed that the children and their parents were not generally willing to have the measurement carried out a second time by the second examiner. This was also the basis for the decision to carry out each measurement only once and not to use the best out of several attempts. The OSMA study has the principle of using a less invasive procedure and an approach that is as child-friendly as possible, which was taken into account throughout the measurement process of MMO in this sub-study.

It can also be viewed critically that the children from the SMA group were examined multiple times and compared to one-off measurements of the healthy controls. The only available comparable data in healthy children of this age group were also collected in a cross-sectional design [18, 19], and current research lacks longitudinal data for children under two years of age.

It is uncertain whether measurements taken on infants and small children who do not actively comply with the request to open their mouths as wide as possible (active MMO) also result in condylar sliding for the maximum possible opening. However, as this applies to all young children, it can be disregarded when comparing groups of that age. When considering the comparability of these measurements to data from older children or adults, it is crucial to differentiate whether the values were obtained through active mouth opening or passive opening (with support). Overall, the entire measurement methodology, including the measuring tools used, must be carefully evaluated to ensure accurate comparability.

## Conclusion

The MMO offers crucial insights into the role of cranial nerve involvement in the progression of SMA, particularly when it comes to disease-modifying therapies. It can be used from birth, and its objective and reliable nature makes it suitable for SMA patient registries.

## Electronic supplementary material

Below is the link to the electronic supplementary material.

Supplementary Material 1

## Data Availability

Data are tabulated in the manuscript. Further data are available from the corresponding author upon reasonable request.
