# Supplementary material for: Maximal mouth opening in infants and toddlers with spinal muscular atrophy: a prospective controlled study
Source: Orphanet J Rare Dis. 2025 Jan 15;20:24. doi: 10.1186/s13023-024-03524-z (PMC11734460; doi:10.1186/s13023-024-03524-z)
Supplement: Supplementary file 1 — Supplementary Material 1 [file 13023_2024_3524_MOESM1_ESM.docx]

| MMO values per age Group | | | | | | | | | | | | | | | |
| --- | --- | --- | --- | --- | --- | --- | --- | --- | --- | --- | --- | --- | --- | --- | --- |
|  | Healthy controls | | |  | SMA total | | |  | SMA (2 *SMN2*) | | |  | SMA (3 *SMN2*) | | |
|  |  | n* | |  |  | n* | |  |  | n* | |  |  | n* | |
| Age Group |  | M (SD) | Mdn (IQR) |  |  | M (SD) | Mdn (IQR) |  |  | M (SD) | Mdn (IQR) |  |  | M (SD) | Mdn (IQR) |
|  |  | 11 | |  |  | 18 | |  |  | 11 | |  |  | 7 | |
| 1 (0-2) |  | 24.09 (4.76) | 25.0 (7) |  |  | 23.44 (4.17) | 24.0 (8) |  |  | 23.0 (4.69) | 26.0 (9) |  |  | 24.14 (3.43) | 22.0 (7) |
|  |  | 12 | |  |  | 4 | |  |  | 2 | |  |  | 2 | |
| 2 (3-5) |  | 28.25 (3.74) | 28.0 (4) |  |  | 24.5 (4.43) | 24.0 (9) |  |  | 28.0 (2.82) | 28.0  (-) |  |  | 21.0 (1.41) | 21.0  (-) |
|  |  | 12 | |  |  | 8 | |  |  | 4 | |  |  | 4 | |
| 3 (6-8) |  | 32.25 (2.52) | 32.5 (4) |  |  | 26.88 (3.79) | 26.0  (5) |  |  | 25.5 (4.5) | 25.5 (9) |  |  | 28.25 (2.87) | 27.5 (5) |
|  |  | 18 | |  |  | 10 | |  |  | 7 | |  |  | 3 | |
| 4 (9-11) |  | 31.78 (3.42) | 32.0 (5) |  |  | 25.9 (2.76) | 26.9 (5) |  |  | 25.29 (3.03) | 24.0 (5) |  |  | 27.33 (1.52) | 27.0  (-) |
|  |  | 11 | |  |  | 6 | |  |  | 4 | |  |  | 2 | |
| 5 (12-14) |  | 34.36 (2.97) | 35.0 (2) |  |  | 25.17 (5.26) | 23.5 (10) |  |  | 23.5 (4.65) | 22.5 (9) |  |  | 28.5 (6.36) | 28.5  (-) |
|  |  | 12 | |  |  | 3 | |  |  | 1 | |  |  | 2 | |
| 6 (15-17) |  | 33.17 (2.44) | 33.5 (5) |  |  | 29.0 (5.19) | 32.0  (-) |  |  | [23]** | [23] |  |  | 32.0 (0) | 32.0 (0) |
|  |  | 15 | |  |  | 15 | |  |  | 11 | |  |  | 4 | |
| 7 (18-24) |  | 31.53 (4.48) | 33.0 (6) |  |  | 27.33 (5.62) | 28.0 (12) |  |  | 25.73 (5.29) | 24.0 (9) |  |  | 31.75 (4.34) | 31.5 (8) |
|  |  | 91 | |  |  | 64 | |  |  | 40 | |  |  | 24 | |
| total |  | 30.9 (4.60) | 32.0  (7) |  |  | 25.66 (4.62) | 26.0 (7) |  |  | 24.7  (4.43) | 24.5  (7) |  |  | 27.3  (4.56) | 27.5  (9) |

**Supplemental Table S1**

Note. MMO=maximal mouth opening, M=mean, SD=standard deviation, Mdn=median, IQR= interquartile range; *n refers to the number of measurements here, ** This individual value cannot represent a measure of central tendency and constitutes a singular observation
